# Insulin resistance unraveled: Hormonal correlations in gestational diabetes

**DOI:** 10.5937/jomb0-49981

**Published:** 2024-11-16

**Authors:** Siyu Chen, Xuru Bai, Xuerui Yin, Lan Bai

**Affiliations:** 1 The First People's Hospital of Chengdu Shuangliu District /Sichuan University West China Airport Hospital, Department of Clinical Laboratory, Chengdu, Sichuan, China

**Keywords:** gestational diabetes mellitus, sex hormonebinding globulin, cortisol, insulin-like growth factor 1, insulin resistance, glucose and lipid metabolism, gestacijski dijabetes melitus, globulin koji vezuje polne hormone, kortizol, faktor rasta 1 sličan insulinu, insulinska rezistencija, metabolizam glukoze i lipida

## Abstract

**Background:**

To investigate the correlation between the variations of cortisol and insulin-like growth factor 1 (IGF1) and sex hormone binding globulin (SHBG) levels with insulin resistance and glucolipid metabolism in gestational glucose diabetics.

**Methods:**

The study included 110 pregnant women diagnosed with gestational diabetes mellitus in the GDM group, and 130 healthy pregnant women in the control group. Data collection, examination of relevant indexes, and comparison of differences in indexes between groups were conducted. Pearson correlation analysis was utilized to identify risk variables associated with GDM development, while binary logistic regression was employed to determine risk factors for GDM development.

**Results:**

The GDM group showed significantly greater levels of FPG, HbA1c, FINS, TG, FFA, Lp-PLA2, HOMA-IR, Cortisol, and IGF-1 compared to the control group (P<0.05), but considerably lower levels of SHBG, and HOMA-β. HOMA-IR was found to be positively correlated with FPG, HbA1c, FINS, TG, FFA, Lp-PLA2, Cortisol and IGF-1, whereas, negatively correlated with SHBG. FINS and SHBG were found to be independent protective factors for GDM (OR=0.463, 0.801, P<0.05), whereas, HbA1c, TG, FFA, and gestational BMI were found to be independent risk factors for GDM (OR=1.992, 4.234, 1.990, 1.629, P<0.05).

**Conclusions:**

SHBG, IGF-1, and Cortisol are all linked to glucose-lipid metabolism indices, and aberrant serum hormone expression is a major contributor to insulin resistance.

## Abstract (sr)

**Uvod:**

Cilj je bio da se istraži korelacija između varijacija kortizola i insulinu sličnog faktora rasta 1 (IGF-1) i nivoa globulina koji vezuje polne hormone (SHBG) sa insulinskom rezistencijom i metabolizmom glukolipida kod gestacijskih dijabetičara glukoze.

**Metode:**

Istraživanjem je obuhvaćeno 110 trudnica sa dijagnozom gestacionog dijabetesa melitusa u GDM grupi i 130 zdravih trudnica u kontrolnoj grupi. Sprovedeno je prikupljanje podataka, ispitivanje relevantnih indeksa i poređenje razlika u indeksima između grupa. Pirsonova korelaciona analiza je korišćena za identifikaciju varijabli rizika povezanih sa razvojem GDM, dok je binarna logistička regresija korišćena za određivanje faktora rizika za razvoj GDM.

**Rezultati:**

GDM grupa je pokazala značajno veće nivoe FPG, HbA1c, FINS, TG, FFA, Lp-PLA2, HOMA-IR, kortizola i IGF-1 u poređenju sa kontrolnom grupom (P<0,05), ali znatno niže nivoe SHBG i HOMA-b. Utvrđeno je da je HOMA-IR u pozitivnoj korelaciji sa FPG, HbA1c, FINS, TG, FFA, Lp-PLA2, kortizolom i IGF-1, dok je u negativnoj korelaciji sa SHBG. Utvrđeno je da su FINS i SHBG nezavisni zaštitni faktori za GDM (OR=0,463,0,801, P<0,05), dok je utvrđeno da su HbA1c,TG,FFA i gestacijski BMI nezavisni faktori rizika za GDM (OR=1,992, 4,234, 1,990, 1,629, P<0.05).

**Zaključak:**

SHBG, IGF-1 i kortizol su svi povezani sa indeksima metabolizma glukoze i lipida, a aberantna ekspresija hormona u serumu je glavni faktor koji doprinosi insulinskoj rezistenciji.

## Introduction

Gestational diabetes mellitus (GDM) affecting approximately 14.8% of pregnant women and infants [1]. The condition is defined by the presence of impaired fasting glucose or impaired glucose tolerance during pregnancy, typically diagnosed between 24–28 weeks of gestation. This condition can result in adverse pregnancy outcomes, including preterm delivery, miscarriage, fetal malformations, neonatal hypoglycemia, and respiratory distress.Sex hormonebinding globulin (SHBG) is a hormone-binding protein that is primarily responsible for regulating hormone activity in the body; decreased SHBG can lead to decreased insulin sensitivity and increased free oestrogen and androgens. This can interfere with insulin signalling and lead to the development of insulin resistance [2] [3]. Insulin-like growth factor 1 (IGF-1) is an insulin-like growth factor involved in mediating cell proliferation and differentiation by binding to the IGF-1 receptor. It is also involved in energy metabolism by regulating the expression of mitochondrial uncoupling protein 2 (UCP2) through the PI3K/AKT/FOXO1 pathway [4]. During pregnancy, the placenta secretes hormones and proteins to adapt to maternal physiology, including insulin resistance and insulin production [5]. The development of gestational diabetes is facilitated by the stimulation of gluconeogenesis and inhibition of glucose utilization in the periphery by cortisol, a glucocorticoid hormone. This process results in hyperglycemia and insulin resistance, primarily through the reduction of glucose transport to the cell membrane by glucose transporter proteins, notably GLUT4 [6]. Although the pathogenesis of GDM is not fully understood. However, β-cell dysfunction and pregnancy-inducedinsulin resistance are two factors contributing to its development, which may lead to impaired insulin secretion [7]. Therefore, early detection and prevention of GDM is crucial to improve its prognosis. The aim of this study was to investigate the correlation between serum SHBG, IGF-1 and cortisol with indices of glucolipid metabolism and insulin resistance in patients with GDM and their impact on the development of GDM.

### Research design and methods

During the period from January 2021 to October 2022, our hospital’s obstetric outpatient clinic performed an obstetric examination. The examination focused on 110 pregnant women who were diagnosed with gestational diabetes mellitus (GDM). These participants were exclusively assigned to the GDM group for this research. The following were the selection criteria: The initial diagnosis of gestational diabetes mellitus (GDM) adhered to the 2020 guidelines outlined by the American Diabetes Association (ADA) and China’s industry diagnostic criteria. The diagnostic process involved conducting a 75 g-oral glucose tolerance test (OGTT) between 24–28 weeks of gestation, with blood glucose levels measured before fasting, at 1 hour, and 2 hours post-glucose intake. Criteria for GDM diagnosis included glucose levels equal to or exceeding 5.1 mmol/L before fasting, 10.0 mmol/L at 1 hour, and 8.5 mmol/L at 2 hours. Inclusion criteria stipulated that GDM would be diagnosed if glucose levels met or exceeded the specified thresholds at any of the designated time points, in the context of a singleton pregnancy and maternal age ranging from 21 to 34 years [3]. Pregnant women lacking pre-pregnancy diabetes mellitus, hypertension, and hyperlipidemia, who received a standardized obstetric evaluation at our institution with comprehensive data.

Exclusion criteria: The following patients were excluded from the study: Patients with complications such as hypertension in pregnancy and intrahepatic cholestasis, those with twin or multiple pregnancies confirmed by ultrasound, individuals with cardiac, hepatic, and renal disease, and those with blood and immune system disorders [3]. Patients with pregnancy complications such as placenta previa, premature rupture of membranes, infections, and amniotic fluid abnormalities, who are not currently prescribed medications that may impact lipid metabolism. The control group included 130 pregnant women with normal OGTT results, excluding those with pregnancy complications and systemic diseases. After enrolling both groups, we measured height and weight, calculated body mass index (BMI), and collected and archived pregnancy and delivery-related data. The study was explained to all subjects and their families, and they all signed an informed consent form. Throughout the treatment process, we strictly adhered to ethical principles and ensured the privacy and safety of the patients.

All research participants provided a 5 mL fasting venous blood sample. After 30 minutes at room temperature, the blood was centrifuged for 10 minutes at 3500 rpm. Detection of glycated hemoglobin (HbA1c) using a fully automated glycated hemoglobin meter from Acuray, Japan. The Beckman automatic biochemistry analyzer (model: AU5800) from the United States was used to measure fasting blood glucose (FPG), triglyceride (TG), total cholesterol (TC), free fatty acid (FFA), high-density lipoprotein (HDL-C), and low-density lipoprotein (LDL-C). Fasting insulin (FINS), lipoprotein-associated phospholipase A2 (Lp-PLA2), and insulin-like growth factor (IGF-1) were measured using the Shenzhen New Industries automatic chemiluminescence instrument (model: MAGLUMI 4000P). Sex Hormone-binding Globulin (SHBG) and Cortisol (Cortisol) were detected by Beckman Automated Chemiluminescence Instrument (model: DXI 800), USA. Insulin resistance index (HOMA-IR) (HOMA-IR = FINS×FPG/22.5) and pancreatic β-cell function index (HOMA-β) (HOMA-β= 20×FINS/(FPG-3.5)) were used to compare various criteria including the number of pregnancies, FPG, HbA1c, FINS, SHBG, body mass index (BMI), and IGF-1 between pregnant women in the GDM group and the control group. Additionally, IGF-1, cortisol, HOMA-IR, HOMA-β, and lipid levels were analyzed.

### Statistical analysis

The clinical data were analysed by applying Statistic Package for Social Science (SPSS) 26.0 statistical software (IBM, Armonk, NY, USA). Normally distributed measurements were expressed as mean ± standard deviation (x̅ ± s), and independent samples t-test was used for comparison between groups. For non-normally distributed data, comparisons between two groups were made using the independent samples non-parametric test, and comparisons between more than two groups were made using the Kruskal- Wallis H(K) test. Pearson correlation was used to analyse the correlation between serum IGF-1, SHBG and Cortisol as well as glycolipid metabolism indexes and HOMA-IR, and binary logistic regression model was used to analyse the risk factors for the development of GDM, and the difference was considered statistically significant at P < 0.05.

## Results

### Comparison of general information between the two groups of study subjects

There was no significant difference in the comparison of the number of pregnancies and births between the two groups of study subjects (P>0.05); the pre-pregnancy BMI, pregnancy BMI, and weight gain during pregnancy of the GDM group were significantly higher than those of the control group (P<0.05), as shown in Table 1.

**Table 1:** Comparison of general information of the two groups of study subjects (x̅±s).

Group	Pregnancy<br>(times)	Delivery<br>(times)	Pre-pregnancy BMI<br>(kg/m^2^)	Pregnancy BMI<br>(kg/m^2^)	Weight gain<br>(kg)
GDM	2.23±1.23	0.47±0.59	22.09±3.29	25.53±3.06	8.67±3.96
Control group	2.30±1.15	0.54±0.59	20.77±3.01	23.49±2.77	6.89±3.37
*t*	-0.47	-0.87	3.23	5.41	3.69
*P*	0.590	0.387	<0.001	<0.001	<0.001

### Comparison of laboratory test indexes between the two groups of study subjects

Compared with the control group, SHBG and HOMA-β levels were significantly lower in the GDM group, and FPG, 1hPG, 2hPG, HbA1c, FINS, HOMA-IR, TG, FFA, Lp-PLA2, IGF-1, and Cortisol levels were significantly higher in the GDM group (P<0.05); there was no significant difference in TC, HDL-C, and LDL-C in both groups (P>0.05), as shown in Table 2.

**Table 2:** Comparison of laboratory test indicators between the two groups of study subjects (x̅±s).

Item Indicator	GDM group	Control group	*t*-value	*P*-value
FPG (mmol/L)	5.27±1.19	4.53±0.25	6.47	<0.001
1hPG (mmol/L)	10.39±1.98	8.07±1.23	11.06	<0.001
2hPG (mmol/L)	8.80±1.88	6.83±1.12	9.64	<0.001
HbA1c (%)	5.36±0.83	4.47±0.20	14.42	<0.001
FINS (uIU/mL)	11.80±4.61	9.89±3.39	3.59	<0.001
HOMA-IR	2.83±1.37	1.99±0.69	5.80	<0.001
HOMA-β	153.38±65.99	205.74±90.87	-5.15	<0.001
TG (mmol/L)	3.51±1.08	2.31±0.85	9.42	<0.001
TC (mmol/L)	5.72±1.14	5.60±1.33	0.74	0.457
HDL-C (mmol/L)	1.82±0.41	1.79±0.38	0.69	0.488
LDL-C (mmol/L)	3.46±0.83	3.44±0.78	0.12	0.906
FFA (mmol/L)	0.77±0.24	0.40±0.12	14.47	<0.001
Lp-PLA2 (ng/mL)	159.10±32.15	114.77±33.48	10.41	<0.001
IGF-1 (ng/mL)	307.27±74.52	222.75±59.82	9.74	<0.001
Cortisol (mmol/L)	719.02±138.85	574.21±125.33	8.49	<0.001
SHBG (nmol/L)	252.21±94.96	379.39±80.13	-11.10	<0.001

### Comparison of clinically relevant indicators in subgroups of HOMA-IR level in GDM group

The GDM group was divided into 3 subgroups according to the tertiles of HOMA-IR level <2.15, 2.15-3.14 and >3.14. With the increase of HOMA-IR value, the levels of FPG, HbA1c, FINS, HOMA-β, TG, FFA, Lp-PLA2, IGF-1, and Cortisol increased, and the level of SHBG decreased, and the difference was statistically significant (P<0.05), as shown in Table 3.

**Table 3:** Comparison of clinical indicators of HOMA-IR level subgroups in GDM group (x̅±s).

Item Indicator	1st quartile group<br>(n=37)	2nd quartile group<br>(n=37)	3rd quartile group<br>(n=36)	*P*-value
Pre-pregnancy BMI (kg/m^2^)	22.30±3.19	22.61±3.62	21.39±2.97	0.202
Pregnancy BMI (kg/m^2^)	25.93±3.18	26.01±3.20	24.76±2.69	0.187
FPG (mmol/L)	4.68±0.49	5.24±0.56	5.92±1.74	<0.001
1hPG (mmol/L)	10.17±1.80	10.57±1.13	10.43±2.73	0.216
2hPG (mmol/L)	8.79±1.27	8.96±1.44	8.63±2.67	0.248
HbA1c (%)	4.97±0.62	5.34±0.83	5.77±0.83	<0.001
FINS (uIU/mL)	6.82±1.67	12.00±1.22	16.70±3.29	<0.001
HOMA-β	129.44±49.54	150.33±43.70	181.13±87.57	<0.001
TG (mmol/L)	2.97±0.88	3.50±0.69	4.07±1.31	<0.001
TC (mmol/L)	5.76±1.03	5.78±1.33	5.61±1.05	0.703
HDL-C (mmol/L)	1.84±0.38	1.79±0.44	1.84±0.41	0.666
LDL-C (mmol/L)	3.42±0.72	3.58±0.95	3.37±0.81	0.679
FFA (mmol/L)	0.60±0.13	0.69±0.17	1.01±0.21	<0.001
Lp-PLA2 (ng/mL)	143.86±20.84	159.61±21.06	174.24±42.84	0.003
IGF-1 (ng/mL)	254.41±57.23	319.93±42.91	354.34±80.93	<0.001
Cortisol (mmol/L)	640.75±124.96	722.87±137.27	795.52±109.93	<0.001
SHBG (nmol/L)	309.89±104.31	246.75±70.70	198.55±72.41	<0.001

### Correlation of serum SHBG, IGF-1, Cortisol and HOMA-IR with glycolipid metabolism indexes in pregnant women in GDM group

In the GDM group, SHBG was negatively correlated with FPG, HbA1c, FINS, TG, FFA, Lp-PLA2 and HOMA-IR (P<0.05); IGF-1 and Cortisol were positively correlated with FPG, HbA1c, FINS, TG, FFA, Lp-PLA2 and HOMA-IR (P<0.05); SHBG, IGF-1 and Cortisol with TC, HDL-C and LDL-C with no correlation (P>0.05), as shown in Table 4.

**Table 4:** Correlation of serum SHBG, IGF-1, Cortisol and HOMA-IR with glycolipid metabolism indexes in pregnant women in GDM group.

Item	SHBG		IGF-1		Cortisol		HOMA-IR	
	*r-value*	*P-value*	*r-value*	*P-value*	*r-value*	*P-value*	*r-value*	*P-value*
FPG	-0.323	0.001	0.487	<0.001	0.403	<0.001	0.675	<0.001
HbA1c	-0.221	0.002	0.208	0.029	0.294	0.002	0.301	0.001
FINS	-0.512	<0.001	0.612	<0.001	0.362	<0.001	0.871	<0.001
TG	-0.352	<0.001	0.354	<0.001	0.349	<0.001	0.577	<0.001
TC	0.162	0.091	-0.031	0.749	-0.157	0.275	-0.112	0.244
HDL-C	0.111	0.249	-0.087	0.365	-0.064	0.504	-0.083	0.388
LDL-C	0.072	0.456	-0.005	0.962	-0.127	0.187	-0.064	0.505
FFA	-0.381	<0.001	0.464	<0.001	0.346	<0.001	0.726	<0.001
Lp-PLA2	-0.284	0.003	0.315	0.001	0.322	0.001	0.454	<0.001
HOMA-IR	-0.567	<0.001	0.711	<0.001	0.419	<0.001	-	-

### Risk factor analysis for GDM

Binary logistic regression was performed with whether pregnant women had gestational diabetes mellitus during pregnancy (assigned value: yes=1, no=0) as the dependent variable, and pre-pregnancy Midgestational Midgestational weight gain, FPG, HbA1c, TG, FINS, FFA, Lp-PLA2, IGF-1, SHBG and Cortisol as the independent variables to analyse the risk factors affecting the development of GDM. Influencing factors, the results showed that increased levels of maternal HbA1c, TG, FFA and maternal BMI during pregnancy were independent risk factors for GDM (P<0.05), while increased levels of FINS and SHBG were independent protective factors for GDM (P<0.05), as shown in Table 5.

**Table 5:** Analysis of risk factors for the development of GDM.

Factors	*B*	*Sb*	*Wald χ^2^*	v	*P*	*OR*	95% Confidence Interval<br>for Overall OR	
							Lower	Upper
Pregnancy BMI	0.488	0.223	4.79	1	0.029	1.629	1.052	2.523
FINS	-0.770	0.254	9.229	1	0.002	0.463	0.282	0.761
TG	1.444	0.552	6.859	1	0.009	4.239	1.438	12.495
HbA1c	0.689	0.260	7.045	1	0.008	1.992	1.198	3.315
SHBG	-0.222	0.100	4.960	1	0.026	0.801	0.658	0.974
FFA	0.688	0.201	11.730	1	0.001	1.990	1.342	2.951

## Discussion

Gestational diabetes mellitus (GDM) is a condition that occurs during pregnancy and is typicallycharacterised by persistent hyperglycaemia [8] [9]. The underlying cause of the disease is insulin resistance and/or defective pancreatic β-cells in the body [10]. GDM can have varying degrees of short- or long-term effects on the health of the pregnant woman, the fetus or the neonate. The development of GDM is associated with a number of risk factors [11], including changes in body mass index, family history of diabetes mellitus, abnormalities in glucose-lipid metabolism, inflammatory response, immune response and insulin resistance. Sex Hormone Binding Globulin is a protein that plays an important role in the human body, mainly binding with sex hormones (such as testosterone, estradiol, etc.) to regulate their concentration and activity in the blood. Many factors can affect the level of sex hormone binding globulin, such as age, gender, endocrine status, etc. The change of its level may be related to some diseases, such as polycystic ovary syndrome, hypogonadism, etc. The primary indicators of insulin resistance include diminished glucose uptake and suppression of hepatic glycogenolysis and peripheral tissue gluconeogenesis. Additionally, it is important to note that insulin is essential for maintaining glucose metabolism homeostasis in skeletal muscle, cardiac muscle, and adipose tissue cells [12].

The results of the present study showed that pregnant women with abnormal glucose metabolism had significantly higher glycaemic and lipid indices than controls, suggesting that GDM is the result of a combination of insulin resistance and/or pancreatic b-cell dysfunction. With the increase in HOMA-IR, the levels of FPG, HbA1c, FINS, TG, FFA, Lp-PLA2, IGF-1, cortisol and HOMA-β also increased, while the level of SHBG decreased, which showed a significant correlation [13]. A study by Yin et al. [14] found that the risk of GDM increased with the elevation of HOMA-IR and HbA1c, and that the likelihood of GDM was significantly increased when both of these factors were elevated. These findings may help in the early identification of women at high risk of GDM in early pregnancy and timely intervention to prevent adverse pregnancy outcomes. This study revealed a significant decrease in HOMA-β levels among patients diagnosed with gestational diabetes mellitus (GDM) compared to controls. Additionally, an increase in HOMA-IR levels was associated with a tendency for higher HOMA-β levels in GDM patients. This phenomenon may be explained by the increased demand for insulin secretion by pancreatic β-cells in GDM patients in response to heightened insulin resistance, leading to fluctuations in blood glucose levels.

The hepatic synthesis of sex hormone binding globulin (SHBG) is integral in the regulation of sexhormones in the circulatory system. Studies indicate that insulin exerts a regulatory influence on SHBG biosynthesis, showing an inverse relationship with insulin resistance. Therefore, SHBG holds potential as a biomarker for gestational diabetes mellitus (GDM) [15]. Studies [16] [17] [18] have indicated that increased insulin levels lower the synthesis of SHBG in the liver. Low SHBG levels are linked to insulin resistance, hyperinsulinaemia, hyperglycaemia and obesity, which are potential predictors of the development of GDM. SHBG may serve as an indicator to monitor the effectiveness of treatment. Moreover, the inhibition of sex hormone binding globulin by glucose or fructose may cause imbalances in sex hormones and glucose-lipid metabolism. This aggravates insulin resistance leading to elevated blood HbA1c levels [19] and subsequent development of gestational diabetes mellitus. Additionally, IGF-1, a growth factor with insulin-like properties, can be produced in the placenta and fetal tissues. It participates in material metabolism regulation through autocrine and endocrine secretion [20]. The compound binds to the insulin receptor, enhancing insulin sensitivity, which is crucial for maintaining normal blood glucose levels during pregnancy and supporting fetal growth and development [21]. Study [22] findings demonstrate that monitoring maternal serum lipocalin and IGF-1 levels during pregnancy can aid in predicting fetal growth and identifying women at risk of GDM. Furthermore, elevated levels of IGF-1 not only enhance the transference of maternal glucose to the fetus and facilitate the production of fats, proteins, and glucose [23]. Furthermore, these mechanisms not only serve to regulate the metabolic processes of the body, but also enhance maternal glucose assimilation, stimulate glycolysis and gluconeogenesis, ultimately resulting in a reduction in blood glucose levels and an amelioration of maternal insulin resistance in individuals with gestational diabetes mellitus (GDM) [24]. Cortisol, a hormone secreted by the adrenocortical bundle, regulates blood glucose levels through hepatic glycogenolysis. Elevated cortisol levels increase insulin antagonism and resistance in skeletal muscle and adipose tissue during pregnancy. A positive correlation was identified between serum cortisol and FPG, INS, and TG in GDM patients, which aligns with the previous findings of Tien Nguyen S et al [25]. Additionally, it was demonstrated [26] that heightened cortisol levels hinder protein synthesis in adipose and extrahepatic tissues, amplify tissue catabolism, and diminish glucose utilisation in peripheral tissues, resulting in an elevation of peripheral tissue glucose. Furthermore, heightened cortisol levels stimulate the liver’s glucose production and hinder insulin-dependent glucose intake, as well as reducing insulin sensitivity in adipose and muscle tissues. These processes lead to insulin resistance, which corroborates the findings of our research.

During the second trimester, there is a rapid increase in the function of the placenta and sex hormones as well as glucocorticoids. This increase peaks between 32–34 weeks, following a rapid increase at 24–28 weeks. The mother secretes more insulin to maintain normal blood glucose levels due to the decrease in insulin sensitivity caused by the increase in hormones, resulting in significant insulin resistance. When insulin secretion is unable to fully counteract insulin resistance, blood glucose levels increase, resulting in the development of gestational diabetes mellitus (GDM). When insulin resistance is low, the pancreatic beta cells can still satisfy the body’s insulin requirement, leading to nearly normal HOMA-β levels. As insulin resistance increases, theremay be a failure of pancreatic β-cells to adequately meet the demands for insulin secretion, resulting in elevated HOMA-β levels. This indicates that the insulin requirements of individuals with gestational diabetes mellitus (GDM) may surpass the functional capacity of pancreatic β-cells, a phenomenon influenced by the degree of insulin resistance. Therefore, comprehensive treatment and management approaches should consider both factors in order to regulate blood glucose levels and mitigate potential negative outcomes during pregnancy.

In conclusion, this study reveals the intricate relationships among hormonal and metabolic factors in the pathogenesis of GDM. While it is possible that elevated levels of specific hormones, such as SHBG, could offer protection against GDM, the primary contributors to the development of GDM are insulin resistance and other metabolic factors, including HbA1c, TG, FFA, and BMI. Furthermore, high levels of FINS may indicate maternal glucose metabolism and disease severity and should be taken into account as a crucial factor in the diagnosis and treatment of GDM. Therefore, personalized treatment plans that address the distinctive metabolic profile of GDM patients could be the optimal approach to managing this disease.

Nonetheless, this study has several limitations. Firstly, being a cross-sectional study, it failed to establish a causal link between changes in serum SHBG, IGF-1, and cortisol concentrations and the onset of GDM. Therefore, additional prospective studies are required to verify these findings. The research project was marred by a limited sample size, as it only included participants from one healthcare facility, possibly restricting its generalizability and introducing potential selection bias. Moreover, the project failed to account for additional confounding factors that could impact the outcomes, including family history of diabetes. Therefore, further comprehensive research iswarranted to explore the relationships between serum levels of sex hormone-binding globulin (SHBG), insulin-like growth factor-1 (IGF-1), and cortisol and gestational diabetes mellitus (GDM) in greater depth. Subsequent examination through larger, multicenter prospective studies is necessary to accurately assess the prognostic value and practical application of these biomarkers.

## Dodatak

### Funding

This study was not supported by any funds.

### Conflict of interest statement

All the authors declare that they have no conflict of interest in this work.

### Contribution

Siyu Chen and Xuru Bai contributed equally to this work.
